# The use of newer anti-seizure medicines in women with epilepsy in pregnancy: A case series

**DOI:** 10.1016/j.ebr.2025.100741

**Published:** 2025-01-15

**Authors:** Joan E. Devin, Fergal O’Shaughnessy, Muskan Sardana, Brian J. Cleary, Jennifer C. Donnelly, Nicola Maher

**Affiliations:** a Irish Medicines in Pregnancy Service, Rotunda Hospital, Parnell Square, Dublin 1, Ireland; b School of Pharmacy and Biomolecular Sciences, Royal College of Surgeons in Ireland, 111 St. Stephen’s Green, Dublin 2, Ireland; c School of Medicine, Royal College of Surgeons in Ireland, 123 St. Stephen’s Green, Dublin 2, Ireland; d Department of Obstetrics and Gynaecology, Rotunda Hospital, Parnell Square, Dublin 1, Ireland

**Keywords:** Women with epilepsy, Newer anti-seizure medicines, Pregnancy, Brivaracetam, Eslicarbazepine, Lacosamide, Zonisamide

## Abstract

Epilepsy is a common serious neurological disorder, affecting approximately 28 per 10,000 pregnancies internationally each year. There are limited data on the use of newer anti-seizure medicines (ASMs) in pregnancy, despite increasing use. We aimed to describe the use of newer ASMs in women with epilepsy (WWE) attending the Rotunda Hospital, Dublin, in pregnancy, between 2018 and 2023. We conducted a retrospective case series using electronic health record data. All WWE with a medication order for a newer ASM and a completed pregnancy were included. We identified 34 pregnancies exposed to newer ASMs, namely zonisamide (35.2 %), brivaracetam (23.5 %), eslicarbazepine (23.5 %), lacosamide (17.6 %), and perampanel (2.9 %). Newer ASMs were used as monotherapy in 58.8 % cases. Levetiracetam was the most commonly prescribed concomitant ASM in polytherapy regimens (32.4 %). Seizures occurred during pregnancy or the postpartum period in 50.0 % and 14.7 % of pregnancies, respectively. Twenty-eight pregnancies (80 %) resulted in a livebirth, with median gestation and birth weight of 39 weeks’ [IQR 2] and 3100 g [IQR 790]. One neonate exposed to polytherapy including eslicarbazepine was observed to have a minor anomaly at birth, not requiring follow-up. Findings show that in WWE, most pregnancies exposed to newer ASMs resulted in healthy livebirths at term without negative outcomes. A high proportion of polytherapy exposures and high rate of seizures during pregnancy suggests that this may be a cohort at greater risk for caesarean section or other complications. Findings should be interpreted with caution, with additional data needed to examine the impact of individual ASMs on outcomes.

## Highlights

• There are limited data on the use of newer antiseizure medicines in pregnancy.
• A case series identified 34 exposed pregnancies in women with epilepsy.
• Exposures included brivaracetam, eslicarbazepine, lacosamide, and zonisamide.
• Most women had a healthy baby at term, with no pattern of neonatal concerns.
• A high rate of polytherapy and high rate of seizures in pregnancy was identified.

## 1 Introduction

Epilepsy is the most common serious neurological disorder seen in pregnant women [1]. It is estimated that more than 15 million women of childbearing potential have epilepsy, with approximately 28 per 10,000 pregnancies affected by epilepsy each year globally [2]. Epilepsy in pregnancy can lead to substantial maternal and neonatal morbidity and mortality, and is one of the most frequent causes of indirect maternal death globally [1], [3]. Seizure control using anti-seizure medicines (ASMs) in pregnancy is key to prevent adverse perinatal outcomes and sudden unexpected death in epilepsy (SUDEP)[1].

ASMs differ in their teratogenic risk. The use of sodium valproate, topiramate, and phenobarbital, in monotherapy and polytherapy, are associated with increased risk of congenital malformations and adverse neurodevelopmental outcomes in infants exposed in pregnancy [3], [4], [5], [6]. Levetiracetam and lamotrigine are considered to be relatively safe during pregnancy [4], [6]. Since the early 2000s newer ASMs have been developed, offering improved seizure control, fewer adverse effects, and improved tolerability for some patients compared with older ASMs [7], [8], [9]. There was a five-fold increase in the use of newer ASMs during pregnancy in the United States between 2001 and 2007 [8], and a trend toward newer ASMs with uncertain risk observed in the Netherlands over a 20-year period [10]. Safety data for most new ASMs are insufficient, and no strong conclusions can yet be made on the safety of newer ASMs in pregnancy, such as brivaracetam, eslicarbazepine, lacosamide, perampanel, and zonisamide [4], [6], [11]. Despite this, newer generation ASMs are being increasingly prescribed for WWE who require polytherapy for seizure control, or for those who cannot tolerate other ASMs due to adverse effects [6], [10], [12].

The Rotunda Hospital is a large maternity hospital in Ireland, with approximately 9,000 births annually. Pregnant women with current or past history of epilepsy are referred to the specialist Obstetric Epilepsy Service antenatally, which has strong links to neurology services in the region [13]. The use of newer ASMs in WWE has not previously been assessed, therefore the aim of this study was to describe the use of newer ASMs in WWE attending the Rotunda Hospital during pregnancy, between 2018 and 2023.

## 2 Methods

### 2.1 Study design

This study was an observational retrospective case series conducted using electronic health record (EHR) data. All WWE attending the Rotunda Hospital for maternity care with a recorded delivery outcome and who had an order for a newer ASM during the study timeframe were included in the study. Newer ASMs were defined as those with marketing authorisation after 2005, a criteria previously used by Hoeltzenbein et al. [12]. ASMs based on these criteria with authorisation for use in Ireland at the time of analysis include: brivaracetam, cannabidiol, cenobamate, eslicarbazepine, fenfluramine, lacosamide, perampanel, rufinamide, stiripentol, and zonisamide. Only women with an antenatal booking appointment and a completed pregnancy up to 2023 were included, representing all women who delivered in our institution since the introduction of the EHR. Completed pregnancies were defined as livebirths and spontaneous miscarriages, where reported by the woman or where hospital care was provided. Women with ongoing pregnancies, or who changed hospital provider during their pregnancy were excluded. The JBI critical appraisal tool for case series was used to inform the study methodology [14] with the Strengthening the Reporting of Observational studies in Epidemiology (STROBE) guidelines used for reporting [15] (Supplementary Data 1 - Appendix A). This study was approved by the Rotunda Hospital Research Ethics Committee (reference: RAG-2024-007).

#### 2.1.1 Data collection

Exposed pregnancies were identified using data extracted from the Maternal and Newborn Clinical Management System (MN-CMS) EHR. Data on women with a recorded diagnosis of epilepsy and who had at least one order for a newer ASM, as defined above, were extracted using validated hospital reports. Maternal demographics (age, sex, ethnicity), clinical characteristics (gravida and parity, obstetric and medical history, seizure activity), and medication orders for ASM and folic acid were retrieved for each exposed pregnancy. Medication orders included both inpatient and outpatient prescriptions, and home medications documented in the medication history. The ILAE 2017 Classification of Seizure Types was used to determine primary type of seizure [16]. Maternal outcomes of interest included pregnancy outcome, delivery type, gestation at delivery, and pregnancy or birth complications. Pregnancy complications included maternal illnesses related to pregnancy such as gestational diabetes mellitus (GDM), severe hyperemesis gravidarum (HEG), pre-eclampsia, infection (including COVID-19), as well as small for gestational age (SGA), defined as a fetus with weight or abdominal circumference < 10th centile [17], or intra-uterine growth restriction (IUGR), defined as a fetus with weight or abdominal circumference < 3rd centile, or < 10th centile with Doppler abnormalities [17].

Birth complications were any problem occurring around the time of delivery, such as cord prolapse, shoulder dystocia, or postpartum haemorrhage. Neonatal outcomes of interest included birth weight, Apgar scores, vitamin K administration, infant feeding type, NICU admission, diagnosis of a congenital malformation, and neonatal complications such as bleeding, hyperbilirubinemia, or sedation and poor feeding after birth. The number of antenatal encounters during each pregnancy, and length of stay at delivery were used as measures of healthcare utilisation. Variables not captured in structured EHR reports were collected using manual chart review. Chart reviews were conducted by JED, MS, and FOS. JED retrieved additional data for all exposed pregnancies, while MS and FOS each retrieved a subset.

### 2.2 Statistical analysis

Use of newer ASMs, demographics and characteristics, healthcare utilisation, and outcomes of exposed pregnancies were analysed descriptively. Interrater reliability was assessed using Fleiss’ kappa. All analyses were performed using R version 4.3.0 [18].

## 3 Results

**Fig. 1:**
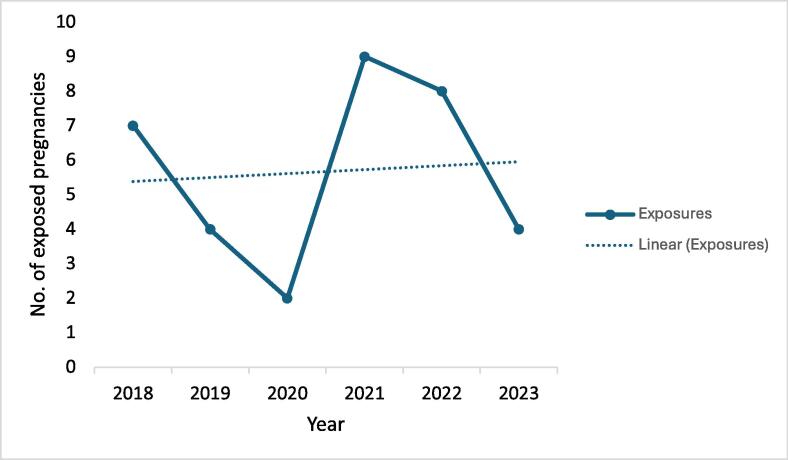
Number of pregnancies exposed to newer ASMs by year. Newer ASMs included brivaracetam, eslicarbazepine, lacosamide, perampanel, and zonisamide. A line chart demonstrates total exposures and linear changes over time between 2018–2023. The highest number of pregnancies exposed to newer ASMs occurred in 2021 (26.5%), while the lowest number occurred in 2020 (5.9 %).

Between 2018 and 2023, there were 34 pregnancies in WWE exposed to one or more newer ASMs, representing 8.1 % of all pregnancies in WWE in the Rotunda Hospital during the study period. Use of newer ASMs varied by year, with most exposures occurring in 2021 (26.4 %) and 2022 (23.5 %) (Fig. 1). The first documented pregnancy exposure to lacosamide occurred in 2021, while all other newer ASMs were observed from 2018.

### 3.1 Newer ASM regimens

The most frequently used newer ASM was zonisamide (35.2 %), followed by brivaracetam (23.5 %), eslicarbazepine (23.5 %), lacosamide (17.6 %), and perampanel (2.9 %), with concomitant exposure to eslicarbazepine and zonisamide in one pregnancy (34 total exposed pregnancies). No exposures to cannabidiol, cenobamate, fenfluramine, rufinamide, or stiripentol were identified. The median maternal age of pregnant women exposed to a newer ASM was 34 years [interquartile range (IQR) 9]. Median gestational age at booking was 13 weeks’ gestation [IQR 3], with 58.8 % of women multiparous at their booking appointment. Most women (85.2 %) received free maternity care through the Irish public healthcare system. The most frequent type of seizures experienced by women taking newer ASMs were generalised onset motor/generalised tonic clonic seizures. Maternal characteristics are summarised in Table 1.

**Table 1:** Maternal demographics and characteristics of pregnancies exposed to newer ASMs.

	All newer ASMs*	BRV	ESL	LCM	ZSN
Exposed pregnancies (n, %)	34 (100)	8 (24)	8 (24)	6 (18)	12 (35)
Age, years (median, [IQR])	34 [9]	36 [7]	38 [15]	26 [15]	34 [6]
Ethnicity, white Irish (n, %)	28 (82)	8 (100)	7	2	10 (83)
Parity at booking (median, min–max)	1 (0–4)	2 (0–3)	1 (0–1)	0 (0–1)	1 (0–4)
Gestation at booking in weeks (median, [IQR])	13 [3]	13 [2]	9 [4]	13 [2]	13 [3]
BMI, kg/m^2^ (median, [IQR])	25 [7]	27 [3]	25 [1]	23 [7]	26 [8]
Primary seizure type (n, %)					
*Generalised onset motor*	15 (44)	6 (75)	2 (25)	0	7 (58)
*Focal to bilateral tonic clonic*	8 (24)	0	3 (38)	2 (33)	3 (25)
*Focal impaired motor/non-motor*	6 (18)	2 (25)	0	4 (67)	0
*Other/unclassified*	6 (18)	0	3 (38)	0	2 (17)
Age at epilepsy diagnosis in years (median, [IQR])	16 [8]	24 [8]	7 [6]	14[12]	16 [4]
Family history epilepsy (n, %)	15 (34)	2 (25)	3 (38)	4 (67)	5 (42)
Current smoker (n, %)	5 (15)	−	−	−	−

Abbreviations: ASMs – antiseizure medicines, BRV – brivaracetam, ESL – eslicarbazepine, LCM – lacosamide, ZSN – zonisamide, IQR – interquartile range.

* Totals column (All newer ASMs) includes 1 perampanel exposure; 1 pregnancy was exposed to both eslicarbazepine and zonisamide (included in individual columns), but is counted as a single pregnancy in the totals column.

**Fig. 2:**
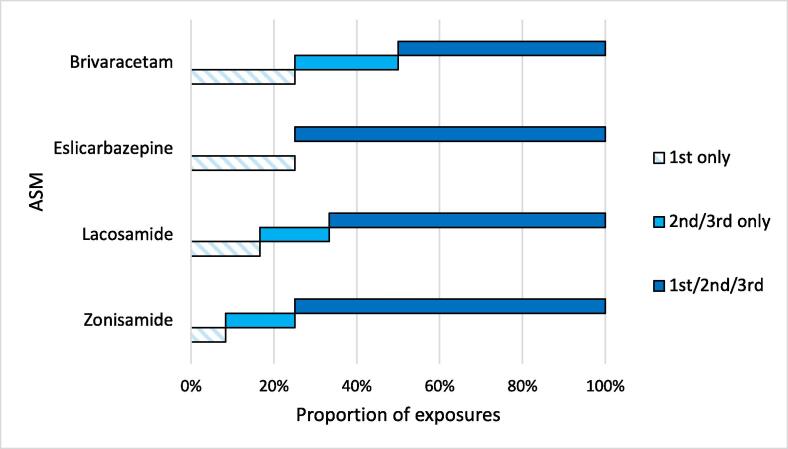
Newer ASM exposure by pregnancy trimester for ASMs with > 1 exposure. The proportion of exposures by trimester is displayed. Where an exposure occurred only in the first trimester, this was a spontaneous miscarriage.

Most pregnancies (67.6 %) were exposed to a newer ASM across all three trimesters (Fig. 2). Where a pregnancy was documented as being exposed to a newer ASM in the first trimester only, this was due to a spontaneous miscarriage occurring. Newer ASMs were used as monotherapy in 20 cases (58.8 %), with monotherapy most prevalent in the brivaracetam-exposed pregnancies (Table 2). A variety of ASMs were used in polytherapy regimens, with levetiracetam the most commonly prescribed concomitant ASM (32.4 %). Other concomitant ASMs included lamotrigine (8.8 %), carbamazepine (2.9 %), pregabalin (2.9 %), and topiramate (2.9 %). Benzodiazepines (clobazam or clonazepam) were prescribed as adjunct treatment in 26.5 % of pregnancies (See Supplementary Data 2 - Appendix B, for distribution of ASM regimens). The majority of women (88.2 %) took folic acid during pregnancy, with most (85.2 %) taking a 5 mg daily dose. There was no exposure to sodium valproate in any of the pregnancies in this case series, and one exposure to topiramate in polytherapy.

**Table 2:** Maternal ASM regimens of pregnancies exposed to brivaracetam, eslicarbazepine, lacosamide, and zonisamide.

	BRV	ESL	LCM	ZSN
Exposed pregnancies (n,%)	8 (23)	8 (23)	6 (17)	12 (34)
Monotherapy (n, %)	7 (88)	4 (50)	3 (50)	5 (42)
Previous alternative ASM reported in clinical documentation (n, %)	6 (75)	2 (25)	5 (83)	5 (42)
Highest daily dose, mg (median, min–max)	150 (50–175)	800 (400–1600)	350 (200–600)	200 (150–400)
No. of concomitant ASMs (median, min–max)	1 (1–3)	2 (1–3)	2 (1–3)	2 (1–4)
Concomitant ASM (n, %) *Levetiracetam* Benzodiazepines* Other**	0 1(13) 1 (13)	2 (25) 3 (38) 1 (13)	2 (33) 2 (33) 0	7 (58) 3 (25) 4 (33)
Therapeutic drug monitoring (n, %)	3 (38)	3 (38)	4 (67)	6 (50)
Folic acid in pregnancy (n, %)	8 (100)	5 (63)	5 (83)	12 (100)
Seizure activity in pregnancy (n, %)	3 (37)	4 (50)	3 (50)	7 (58)
Seizure activity postpartum (n, %)	1 (13)	1 (13)	2 (33)	1 (8)
GTC seizures (n, %)	2 (25)	3 (38)	1 (17)	4 (33)

Abbreviations: ASM – antiseizure medicine, BRV – brivaracetam, ESL – eslicarbazepine, GTC – generalised tonic clonic, LCM – lacosamide, ZSN – zonisamide, IQR – interquartile range.

*Benzodiazepines include clobazam and clonazepam. **Other ASMs include lamotrigine, carbamazepine, pregabalin, and topiramate. > 1 concomitant ASM is possible.

Half of all women experienced seizure activity during pregnancy, with 14.7 % experiencing seizure activity postpartum. A higher proportion of seizures occurred in women taking ASM polytherapy; seizures occurred in 78.6 % of pregnancies exposed to ASM polytherapy, while seizures occurred in 40.0 % of pregnancies exposed to ASM monotherapy. Tiredness, diarrhoea, poor compliance with ASM regimen, and increased dose requirements as the pregnancy progressed were identified as provoking factors for seizure activity.

### 3.2 Maternal and neonatal outcomes

Twenty-eight (80 %) pregnancies exposed to newer ASMs resulted in a livebirth, with one multiple birth. Spontaneous miscarriages occurred in pregnancies exposed to all newer ASMs, except perampanel. Of the spontaneous miscarriages, three occurred before 12 weeks of pregnancy, one occurred at 14 weeks, and gestation for two was unknown.

Spontaneous labour occurred in 32 % of pregnancies. Most (57 %) pregnancies were delivered by caesarean section. Maternal outcomes are summarised in Table 3.

**Table 3:** Maternal pregnancy and birth outcomes of pregnancies exposed to newer ASMs.

	All newer ASMs*	BRV	ESL	LCM	ZSN
Exposed pregnancies (n,%)	34 (100)	8 (23)	8 (23)	6 (17)	12 (34)
Pregnancy outcome (n, %)					
*Livebirth*	28 (82)	6 (75)	6 (75)	5 (83)	11 (92)
*Spontaneous miscarriage*	6(18)	2 (25)	2 (25)	1 (17)	1 (8)
Gestation at birth in weeks^a^ (median, min–max)	39 (27–41)	39 (27–40)	39 (37–41)	38 (34–39)	39 (36–40)
Onset of labour^a^ (n, %)					
*Spontaneous*	9 (32)	1 (17)	0	3 (60)	5 (46)
*Induced*	6 (21)	1 (17)	1 (17)	0	3 (27)
*Pre-labour LSCS*	13 (46)	4 (66)	5 (83)	2 (40)	3 (27)
Mode of delivery^a^ (n, %)					
*SVD/OVD*	12 (43)	2 (33)	0	3 (60)	6 (55)
*LSCS*	16 (57)	4 (66)	6 (100)	2 (40)	5 (46)
Pregnancy complications^b^					
*GDM*	4 (12)	2 (25)	0	1 (17)	1 (8)
*Antepartum bleeding*	4 (12)	0	2 (25)	0	2 (17)
*Infection*	5 (15)	0	2 (25)	1 (17)	2 (17)
*Other*^c^	4 (12)	2 (25)	1 (13)	1 (17)	0
No. of antenatal patient encounters^a^ (median, [IQR])	22 [13]	23 [6]	19 [3]	24 [9]	22 [8]
Maternal LOS at delivery in days^a^ (median, [IQR])	3 [3]	3 [3]	5 [4]	4 [2]	2 [2]

Abbreviations: ASM – antiseizure medicine, BRV – brivaracetam, ESL – eslicarbazepine, GDM – gestational diabetes mellitus, LCM – lacosamide, ZSN – zonisamide, IQR – interquartile range, SVD – spontaneous vaginal delivery, OVD – operative vaginal delivery, LSCS – lower segment caesarean section, LOS – length of stay.

* Totals column (All newer ASMs) includes 1 perampanel exposure; 1 pregnancy was exposed to both eslicarbazepine and zonisamide (included in individual columns), but is counted as a single pregnancy in the totals column.

a Excludes spontaneous miscarriages.

b Women could contribute > 1 pregnancy complication.

c Severe hyperemesis gravidarum, severe anaemia, or substance misuse.

Pregnancy or birth complications were reported in 22 (64.7 %) pregnancies. Infection in pregnancy was the most common complication, seen in 14.7 % of all included pregnancies (Table 3). Gestational diabetes was present in 25 % of brivaracetam-exposed pregnancies, 16.7 % of lacosamide-exposed pregnancies, and 8.3 % of zonisamide-exposed pregnancies. A non-substantial antepartum haemorrhage was reported by 25 % of women taking eslicarbazepine, and 16.7 % of women taking zonisamide (Table 3).

Birth complications were infrequent, with one instance of shoulder dystocia, and no reported postpartum haemorrhages. Antenatally, social challenges were also present in 20.6 % of pregnancies in this study, including substance misuse, domestic abuse, and deprivation or homelessness.

The median gestational age at birth was 39 weeks’ gestation [IQR 2], with median birth weight 3100 g [IQR 790]. The lowest median birth weight and gestational age was observed in neonates exposed to lacosamide in utero. The majority (82.6 %) of neonates exposed to newer ASMs in this study had a weight within normal range[17], between the 10th and 90th centile for their gestation.

Most neonates (89.7 %) did not require admission to the Neonatal Intensive Care Unit (NICU). There were no NICU admissions of neonates exposed to zonisamide in utero. Reasons for NICU admission included prematurity (3.4 %), resuscitation at birth (3.4 %), and jaundice requiring phototherapy (3.4 %).

Hyperbilirubinemia (jaundice) was the most common neonatal problem, reported in 37.9 % of all neonates after birth. The highest proportion was seen in neonates exposed to zonisamide, with 41.7 % of babies developing jaundice before discharge, although all were mild cases. Poor feeding or gastrointestinal disturbances were reported in two (33.3 %) brivaracetam and eslicarbazepine-exposed neonates, and one neonate (8.3 %) exposed to zonisamide. Sedation was less commonly seen, although two neonates (40 %) exposed to lacosamide had increased sedation, subsequently causing poor feeding.

All neonates received intramuscular vitamin K shortly after birth. Almost half (48 %) were breastfeeding at discharge from hospital. Neonatal outcomes are summarised in Table 4.

**Table 4:** Outcomes of neonates exposed to newer ASMs in utero.

	All newer ASMs*	BRV	ESL	LCM	ZSN
Neonates (n, %)^a^	29 (100)	6 (21)	6 (21)	5 (17)	12 (41)
Sex, female (n, %)	16 (55)	5 (83)	−	−	5 (42)
Gestation at birth in weeks^a^ (median, min–max)	39 (27–41)	39 (27–40)	39 (37–41)	38 (34–39)	39 (36–40)
Birth weight, g (median, [IQR])	3100 [790]	3230 [995]	3100 [457]	2660 [550]	3150 [520]
SGA/IUGR (n, %)	4 (14)	2 (33)	0	1 (20)	1 (8)
Apgar scores (median, min–max)					
*1* min	9 (3–10)	9 (7–10)	9 (3–9)	9 (9–9)	9 (8–10)
*5* min	10 (8–10)	10 (8–10)	10 (9–10)	10 (10–10)	10 (10–10)
NICU admission (n, %)	3 (10)	1 (17)	1 (17)	1 (20)	0
Neonatal problems^b^					
*Hyperbilirubinemia*	11 (38)	2 (33)	2 (33)	2 (40)	5 (42)
*Poor feeding/GI disturbance*	7 (24)	2 (33)	2 (33)	2 (40)	1 (8)
*Sedation*	2 (7)	0	0	2 (40)	1 (8)
Neonate LOS in days (median, [IQR])	3 [3]	3 [3]	5 [4]	4 [2]	3 [1]
Breastfeeding (n, %)	14 (48)	3 (50)	3 (50)	4 (80)	4 (33)

Abbreviations: ASM – antiseizure medicine, BRV – brivaracetam, ESL – eslicarbazepine, LCM – lacosamide, ZSN – zonisamide, IQR – interquartile range, IUGR – intrauterine growth restriction, LOS – length of stay, NICU – neonatal intensive care unit.

* Totals column (All newer ASMs) includes 1 perampanel exposure; 1 pregnancy was exposed to both eslicarbazepine and zonisamide (included in individual columns), but is counted as a single pregnancy in the totals column.

a Includes 1 twin pregnancy.

b Multiple neonates had > 1 concurrent problem.

Two neonates, both exposed to ASM polypharmacy in utero, had IUGR [17]. The first, who was exposed to eslicarbazepine, pregabalin, and benzodiazepines in pregnancy, was delivered in poor condition and required advanced resuscitation at birth. The second neonate was exposed to zonisamide, carbamazepine, and levetiracetam polytherapy, and had no other reported problems. A further two neonates were preterm and SGA. The first was exposed to brivaracetam monotherapy, while the second was exposed to lacosamide monotherapy. One neonate exposed to brivaracetam was born large for gestational age, but this was a pregnancy complicated by GDM.

One neonate, exposed to eslicarbazepine and levetiracetam in utero, was observed to have a minor anomaly at birth (not requiring reporting to EUROCAT as per classification guide [19]). Two neonates were referred to paediatric follow-up for developmental delay within the first year of life. One neonate was exposed to eslicarbazepine polytherapy. The second was exposed to brivaracetam monotherapy, and was born very preterm for maternal obstetric indications. It is important to note that these neonatal outcomes were incidental observations, and that outcomes beyond the neonatal period would not be reliably captured or routinely contained in our dataset.

### 3.3 Interrater reliability

Fleiss’ kappa demonstrated good agreement between chart reviewers, κ = 0.791 (95 % CI, 0.786–0.793), p < 0.001.

## 4 Discussion

Between 2018 and 2023, there were 34 pregnancies exposed to the newer ASMs brivaracetam, eslicarbazepine, perampanel, lacosamide, or zonisamide in the Rotunda Hospital. No exposures to cannabidiol, cenobamate, fenfluramine, rufinamide, or stiripentol were identified.

This case series demonstrated a low, but relatively consistent use of newer ASMs in WWE. There were more exposures to zonisamide than any other newer ASM. Newer ASMs were used as monotherapy in 58.8 % of pregnancies. Where there was polytherapy, a variety of ASMs were used, with levetiracetam the most commonly prescribed concomitant ASM. Benzodiazepines were prescribed as adjunct treatment in 26.5 % of pregnancies, but it was not possible to estimate duration of exposure. As benzodiazepines may be used for seizure cover if ASM levels are low or if changing therapy, these prescriptions may reflect episodic use. There were no concomitant exposures to sodium valproate during pregnancy, and one exposure to topiramate, which occurred before the introduction of the Topiramate Pregnancy Prevention Programme [20].

### 4.1 Pregnancy outcomes

The background rate of miscarriage in the general population is between 10 and 30 % [21], [22]. Twenty-five percent of pregnancies exposed to brivaracetam or eslicarbazepine resulted in a spontaneous miscarriage, a marginally higher proportion than the overall rate of 18 % identified in the case series, but again in line with the background rate of miscarriage. The rate of miscarriage in the general population increases with increasing maternal age [23], [24]. A Danish population-based study of fetal loss in 634,272 women (1,221,546 pregnancy outcomes) found that miscarriage rate in women aged 35–39 was 24.6 %, increasing to 93.4 % in women ≥ 45 years [24]. The median age of women exposed to brivaracetam or eslicarbazepine in pregnancy was higher than those exposed to lacosamide or zonisamide, so age may be a contributory factor.

There was no apparent elevated occurrence of induction of labour or preterm labour in exposed pregnancies. Approximately 8 % of babies in this case series were born preterm, which is similar to the background rate in Ireland of approximately 7 % [25], and less than the estimated 10 % that occur worldwide annually [26]. An overall caesarean section rate of 57.1 % was observed, which is higher than the hospital average of 39 %, reported in 2023. Rates of caesarean section appear to vary in WWE, with hospital cultural factors and higher income identified as more likely contributory factors than patient variables in a recent review [6]. In this case series the caesarean section rate appeared to be driven by obstetric and clinical need. Maternal reasons for caesarean section included worsening seizures (31.3 %), pre-eclampsia or infection (12.5 %), or elective for previous obstetric anal sphincter injury or caesarean section (37.5 %). Fetal reasons included non-reassuring CTG and IUGR (12.5 %).

### 4.2 ASM use and seizures in pregnancy

Previous alternative ASM therapy and polytherapy was reported across all groups, highlighting the role of newer ASMs as add-on treatment for focal or partial seizures and refractory epilepsy [12]. In contrast, brivaracetam monotherapy was more common. Almost all (87.5 %) women who were taking brivaracetam had a mental health condition, and were likely taking brivaracetam due to its more favourable psychiatric profile than its sister drug levetiracetam [27], [28]. Experiencing a low mood with levetiracetam is the primary reason for switching to brivaracetam in our institution.

Seizures occurred in 78.6 % of pregnancies exposed to ASM polytherapy, while seizures occurred in 40 % of pregnancies exposed to ASM monotherapy. Seizures in the year prior to conception is a key risk factor for seizures in pregnancy [29], and seizure activity was reported in the year prior to pregnancy by 78.9 % women who went on to have seizures during pregnancy or the postpartum period. Frequent contact with the hospital was evident, with 57.1 % of pregnancies that resulted in a livebirth having regular therapeutic drug monitoring. While therapeutic drug monitoring is routine for older ASMs in our institution, this low level is likely due to having no local access to a therapeutic monitoring service for these newer ASMs during the study period. As of 2024, the hospital has access to therapeutic drug monitoring for all ASMs through a UK laboratory.

The median number of antenatal encounters (which encompassed all antenatal visits, ultrasound scans, emergency room visits, antenatal class, or any other type of outpatient appointment) for all pregnancies exposed to newer ASM was 22 encounters [IQR 13]. The number of antenatal encounters per pregnancy was similar for all newer ASMs. Median length of stay at delivery for all pregnancies was three days [IQR 3]. Length of stay for pregnancies exposed to eslicarbazepine was five days [IQR 4], but this was related to mode of delivery, as all women in this group had a caesarean section.

### 4.3 Neonatal outcomes

A number of previous studies have raised concerns about the potential teratogenicity of zonisamide[30], [31], and its association with fetal growth restriction and low birth weight [30], [32]. Although no particular safety problems have been linked to eslicarbazepine use in pregnancy, some studies have identified possible relationships between exposure and spontaneous miscarriage or congenital anomalies[11], [33]. Available information on lacosamide does not suggest that it is a major teratogen [7], [11]. There was no observed increase in preterm birth, diagnosis of IUGR, major congenital anomalies, or other adverse perinatal outcomes in neonates exposed in utero to newer ASMs in this case series. One minor congenital anomaly was observed in an infant exposed to eslicarbazepine and levetiracetam. No neural tube defects (NTDs) or other major anomalies occurred in infants exposed to any of the newer ASMs in this case series. However, as a descriptive case series with a limited sample, it is not possible to draw conclusions on outcomes. Despite this, from a clinical perspective, these data add to existing case studies of newer ASM use in pregnancy, which together may be of value in helping prescribers and WWE make risk–benefit decisions on their use in pregnancy.

### 4.4 Folic acid use

Folic acid is recommended in pregnancy for all women, due to its role in the prevention of NTDs[34], [35]. There was good use of folic acid in this case series. In the 28 pregnancies that resulted in a livebirth, all but one reportedly took folic acid at some point in their pregnancy (96.4 %). Twelve of the women were taking folic acid pre-conceptually. A 5 mg folic acid once daily dose was the most frequent dose prescribed (93.5 %). In 2023, one woman took 5 mg folic acid in the first trimester, with her dose reduced to 400mcg after 12 weeks’ gestation. This pattern was not observed in any other pregnancy.

Concerns have been raised about the potential impact of high-dose folic acid in pregnancy. A large observational cohort study of registry data from three Nordic countries, published in 2022, found that antenatal exposure to high-dose folic acid (≥1mg daily) in pregnancy in WWE, was associated with an increased risk of cancer in their children [36]. Risk did not differ by ASM type [36]. However, this study was not able to determine a causal relationship between antenatal folic acid use and the development of childhood cancers in children born to WWE [36], and further studies are needed to determine whether a causal relationship exists. While there is ongoing debate about the most appropriate dose of folic acid for WWE [37], [38], there does not yet appear to be a change in practice in relation to the prescribing of high-dose folic acid in our cohort.

### 4.5 Epilepsy in pregnancy data

From the late 1960′s, evidence was emerging about the potential teratogenic effects of sodium valproate, with ‘Foetal Valproate Syndrome’ first described in 1984 [39]. It was not until the advent of prospective pregnancy registries that the magnitude of the risk and evidence of harm became fully understood [39], [40]. Considering the history of valproate, it is essential to address the absence of information in relation to newer ASM use in pregnancy.

In 2020, ‘First Do No Harm − the report of The Independent Medicines and Medical Devices Safety Review', was published in the United Kingdom (UK) [39]. This review examined how the UK healthcare system responds to reports about harmful side effects from medicines and medical devices, and considered how to respond to them more quickly and effectively in the future [39]. In relation to sodium valproate, one of the actions recommended in the review was the development of a registry for all women taking ASMs who become pregnant, to include longitudinal mandatory reporting of data relating to them and their children [39]. In the absence of a mandatory Irish epilepsy in pregnancy registry, routinely collected EHR data may be an alternative, comprehensive data source. The MN-CMS EHR is currently implemented in four maternity units in Ireland, encompassing 40 % of births annually (soon to be 65 % of all births). MN-CMS data can contribute to the body of knowledge on ASM use in pregnancy in Ireland, with real-time insight into national prescribing practices and outcomes increasingly possible as more maternity units implement the EHR. As women with poor response to known safe medications often rely on newer ASMs for seizure control, collection of this data and further research is imperative to facilitate shared decision-making with women for whom seizure control is particularly challenging and for whom pregnancy poses a high-risk period.

### 4.6 Strengths and limitations

To our knowledge, this is one of the largest case series of newer ASMs to date, with data on 34 pregnancies exposed to brivaracetam, eslicarbazepine, lacosamide, perampanel, or zonisamide. This study demonstrates the value of using routinely collected EHR for research on use of ASM in pregnancy, with the potential to replicate this study in other MN-CMS sites and gain insight into newer ASM use and changing practices at a population level.

EHR data permits analysis of large retrospective data sources, while avoiding the potential reporting bias of retrospective registries in relation to adverse perinatal outcomes. Pregnancies for inclusion were identified using structured data fields from inpatient and outpatient prescriptions for newer ASMs, and home medications documented in the medication history. There was potential to capture medication exposure at various time points throughout the pregnancy, including booking interviews, antenatal appointments, appointments with the Epilepsy Advanced Nurse Practitioner, and medicines reconciliation at antenatal and delivery admission and discharge, therefore ascertainment of ASM orders in the study was likely high.

One limitation of using EHR data is limited or missing patient fields, as data are collected for routine clinical or billing use as opposed to use in research. As we used manual chart reviews as well as structured reports, we were able to retrieve additional patient information contained within narrative notes or scanned documents and address this limitation.

Completed pregnancies in this study included livebirths, and spontaneous miscarriages where reported by the woman or where hospital care was provided. It was not possible to include women who had a spontaneous miscarriage or an elective termination prior to seeking antenatal care, and so the use of newer ASM in this cohort is unclear. In Ireland, termination of pregnancy can occur on request up to 12 weeks of pregnancy, but later in cases only where the woman's life or health is at risk or when the fetus has a fatal abnormality. We did not identify any terminations in this study.

Despite the fact that this is a relatively large case series, the overall low number of newer ASMs included in the study means that it is not possible to draw firm conclusions from the data.

## 5 Conclusions

There was low, but consistent use of newer ASMs in WWE attending the Rotunda Hospital between 2018 and 2023. Most pregnancies exposed to newer ASMs resulted in healthy livebirths at term, with no significant concerns identified. Overall, while this case series indicates that women taking newer ASMs do not appear to be at high risk for negative outcomes, the high rate of seizures during pregnancy and high percentage of polytherapy exposures suggests that this may be a cohort at greater risk for caesarean section or other complications. Findings should be interpreted with caution due to the limited number of exposures, multiple observed variables, and retrospective nature of the study, with additional data and further research needed to examine the impact of individual ASMs on maternal and neonatal outcomes.

## 6 Statement on the use of Generative AI and Figures, images and artwork

All authors declare that generative AI has not been used to produce any part of this manuscript or figures/images.

## 7 Funding information

This research did not receive any specific grant from funding agencies in the public, commercial, or not-for-profit sectors.

## 8 Ethical approval.

This study was approved by the Rotunda Hospital Research Ethics Committee (reference: RAG-2024-007).

## 9 Data sharing statement

The data that support the findings of this study are not publicly available due to privacy and ethical reasons.

## CRediT authorship contribution statement

**Joan E. Devin:** Writing – original draft, Validation, Investigation, Formal analysis, Data curation, Conceptualization. **Fergal O’Shaughnessy:** Writing – review & editing, Validation, Investigation, Conceptualization. **Muskan Sardana:** Writing – review & editing, Investigation. **Brian J. Cleary:** Writing – review & editing, Conceptualization. **Jennifer C. Donnelly:** Writing – review & editing, Conceptualization. **Nicola Maher:** Writing – review & editing, Conceptualization.

## Declaration of competing interest

The authors declare that they have no known competing financial interests or personal relationships that could have appeared to influence the work reported in this paper.

## Glossary

ASM: Anti-Seizure Medicine
BRV: Brivaracetam
CTG: Cardiotocograph
EHR: Electronic Health Record
ESL: Eslicarbazepine
EUROCAT: European Concerted Action on Congenital Anomalies and Twins
GI: Gastrointestinal
ILAE: International League Against Epilepsy
IQR: Interquartile Range
IUGR: Intrauterine Growth Restriction
LCM: Lacosamide
LOS: Length of Stay
LSCS: Lower Segment Caesarean Section
MN-CMS: Maternal and Newborn Clinical Management System
NICU: Neonatal Intensive Care Unit
NTD: Neural Tube Defect
OVD: Operative Vaginal Delivery
STROBE: Strengthening the Reporting of Observational Studies in Epidemiology
SUDEP: Sudden Unexpected Death in Epilepsy
SVD: Spontaneous Vaginal Delivery
WWE: Women with Epilepsy
ZSN: Zonisamide
