# Supplementary material for: The use of newer anti-seizure medicines in women with epilepsy in pregnancy: A case series
Source: Epilepsy Behav Rep. 2025 Jan 15;29:100741. doi: 10.1016/j.ebr.2025.100741 (PMC11804773; doi:10.1016/j.ebr.2025.100741)
Supplement: Supplementary Data 2 [file mmc2.docx]

**Appendix B**

| Table B1.  Distribution of antiseizure medicine regimens in pregnancies exposed to newer ASM (n=34). | | |
| --- | --- | --- |
| ASM | **n** | **%** |
| Brivaracetam | 7 | 20.6 |
| Zonisamide | 5 | 14.7 |
| Eslicarbazepine | 4 | 11.7 |
| Lacosamide | 3 | 8.8 |
| Eslicarbazepine + Levetiracetam + Clobazam | 2 | 5.9 |
| Zonisamide + Levetiracetam + Clobazam | 2 | 5.9 |
| Brivaracetam + Lamotrigine + Clobazam | 1 | 2.9 |
| Eslicarbazepine + Pregabalin + Clobazam | 1 | 2.9 |
| Eslicarbazepine + Zonisamide | 1 | 2.9 |
| Lacosamide + Clonazepam | 1 | 2.9 |
| Lacosamide + Levetiracetam | 1 | 2.9 |
| Lacosamide + Levetiracetam + Clobazam | 1 | 2.9 |
| Perampanel | 1 | 2.9 |
| Zonisamide + Levetiracetam + Lamotrigine | 1 | 2.9 |
| Zonisamide + Levetiracetam + Topiramate + Clobazam | 1 | 2.9 |
| Zonisamide + Levetiracetam+ Carbamazepine | 1 | 2.9 |
| Zonisamide + Lamotrigine | 1 | 2.9 |
